# Mental imagery of speech: linking motor and perceptual systems through internal simulation and estimation

**DOI:** 10.3389/fnhum.2012.00314

**Published:** 2012-11-28

**Authors:** Xing Tian, David Poeppel

**Affiliations:** Poeppel Lab, Department of Psychology, New York UniversityNew York, NY, USA

**Keywords:** internal forward model, efference copy, corollary discharge, sensory-motor integration, mirror neurons, auditory hallucination, stuttering, phantom limb

## Abstract

The neural basis of mental imagery has been investigated by localizing the underlying neural networks, mostly in motor and perceptual systems, separately. However, how modality-specific representations are top-down induced and how the action and perception systems interact in the context of mental imagery is not well understood. Imagined speech production (“articulation imagery”), which induces the kinesthetic feeling of articulator movement and its auditory consequences, provides a new angle because of the concurrent involvement of motor and perceptual systems. On the basis of previous findings in mental imagery of speech, we argue for the following regarding the induction mechanisms of mental imagery and the interaction between motor and perceptual systems: (1) Two distinct top-down mechanisms, *memory retrieval* and *motor simulation*, exist to induce estimation in perceptual systems. (2) Motor simulation is sufficient to internally induce the representation of perceptual changes that would be caused by actual movement (perceptual associations); however, this simulation process only has modulatory effects on the perception of external stimuli, which critically depends on context and task demands. Considering the proposed simulation-estimation processes as common mechanisms for interaction between motor and perceptual systems, we outline how mental imagery (of speech) relates to perception and production, and how these hypothesized mechanisms might underpin certain neural disorders.

## Introduction

Mental imagery can be characterized as a quasi-perceptual experience, induced in the absence of external stimulation. Neuroimaging studies have shown that common neural substrates mediate mental imagery and the corresponding perceptual processes, such as in visual (e.g., Kosslyn et al., 1999; O'Craven and Kanwisher, 2000), auditory (e.g., Zatorre et al., 1996; Kraemer et al., 2005), somatosensory (e.g., Yoo et al., 2003; Zhang et al., 2004), and olfactory domains (e.g., Bensafi et al., 2003; Djordjevic et al., 2005). The demonstration of activation in corresponding perceptual regions during mental imagery has provided strong evidence to support the claim that the perceptual experience during mental imagery is mediated by modality-specific neural representations (see the review by Kosslyn et al., 2001). However, the top-down “induction mechanism” for the neural activity mediating mental imagery is not well understood.

We focus here on the role of the motor system in the construction of perceptual experience in mental imagery. We propose a motor-based mechanism that is an alternative (additional) mechanism to Kosslyn's memory-attention-based account (Kosslyn, 1994, 2005; Kosslyn et al., 1994): planned action is simulated in motor systems to internally derive the representation of perceptual changes that would be caused by the actual action (perceptual associations). We suggest that the deployment of these two distinct mechanisms depends on task demands and contextual influence. Studies of mental imagery of speech are summarized to provide evidence for the proposed account—and for the coexistence of both mechanisms. We discuss the motor-to-sensory integration process and propose some working hypotheses regarding certain neural and neuropsychiatric disorders from the perspective of the proposed internal simulation and estimation mechanisms.

## Different routes for inducing mental images

### Mental imagery of perception as memory retrieval (direct simulation)

Mental imagery has been proposed to be essentially a memory retrieval process. That is, perceptual experience is simulated by reconstructing stored perceptual information in modality-specific cortices (Kosslyn, 1994, 2005; Kosslyn et al., 1994). In particular, the process, guided by attention, retrieves object and spatial properties stored in long-term memory to reactivate the topographically organized sensory cortices that represent the object features. Through top-down (re)construction of the neural representation that is similar to the result of bottom-up perceptual processes, the perceptual experience can be re-elicited without the presence of any physical stimuli during mental imagery. This attention-guided memory retrieval process has been demonstrated, for example, in the visual imagery of faces (Ishai et al., 2002).

Mental imagery is further hypothesized to be a predictive process (for future perceptual states), in which the dynamics of perceptual experience can be retrieved/calculated and reconstructed internally (Moulton and Kosslyn, 2009). That is, given an initial point, the series of future perceptual states can be internally simulated by following the regularity (temporal and causal constraints) stored in declarative memory (general knowledge). The mapping between internal simulation and the perception of external stimulation is thought not to be necessarily isomorphic (Goldman, 1989), as only the essential intermediate states are required to have a one-to-one mapping (Fisher, 2006). Because this proposed simulation process is executed entirely within perceptual domains on the basis of memory retrieval—without any representational transformation between motor and perceptual systems—we refer to this account as *direct simulation*.

### Mental imagery of motor action as estimation deriving from simulation

Motor imagery is thought to be the process that internally simulates planned actions, by activating similar neural substrates that mediate motor intention and preparation (Jeannerod, 1995, 2001; Decety, 1996). Numerous studies have demonstrated both frontal and parietal activity during motor imagery (Decety et al., 1994; Lotze et al., 1999; Gerardin et al., 2000; Ehrsson et al., 2003; Hanakawa et al., 2003; Dechent et al., 2004; Meister et al., 2004; Nikulin et al., 2008). However, motor system activation does not necessarily link to the kinesthetic feeling generated during motor imagery. The residual neural activity, resulting from the absence of external somatosensory feedback, is thought to mediate the kinesthetic experience during motor imagery (Jeannerod, 1994, 1995). The implicit assumption of the “residual activity account” is that the internal motor simulation during imagery should be transformed into the same representational format as the one resulting from somatosensory feedback. That is, the somatosensory consequences of motor simulation should be estimated. This is consistent with the view that parietal rather than frontal motor regions mediate motor awareness (Desmurget and Sirigu, 2009). In support of the claim that parietal regions mediate somatosensory estimation, direct current stimulation over parietal cortex induces false belief of movement (Desmurget et al., 2009); parietal lesions also impaired the temporal precision of performing motor imagery tasks (Sirigu et al., 1996). Cumulatively, the results suggest that motor simulation in frontal cortex converges in parietal regions to form a kinesthetic representation.

The internal transformation between motor simulation and somatosensory estimation has been proposed in the context of internal forward models in the motor control literature [see the review by Wolpert and Ghahramani (2000)]. The core presupposition is that the neural system can predict the perceptual consequences by internal simulating a copy of a planned action command (the efference copy). Mental imagery has been linked to the concept of internal forward models by the argument that the subjective feeling in mental imagery is the result of the internal estimation of the perceptual consequences following the internal simulation of an action (Grush, 2004). Consistent with this hypothesis, we propose here that the kinesthetic feeling in motor imagery is the result of somatosensory estimation, derived from internal simulation that closely mimics the dynamics of a motor action. We refer to this account as *motor simulation and estimation*.

The *motor simulation and estimation* account differs from the *direct simulation* (memory retrieval) account in that it requires a transformation between motor and somatosensory systems. Our question here, though, extends beyond this: can a motor simulation deliver perceptual consequences that extend to other sensory domains (such as visual and auditory) as well? If so, internal simulation and estimation processes would serve as an additional path to induce modality-specific neural representations similar to the ones induced on the basis of memory retrieval. In the next section, we discuss this possibility in the framework of internal forward models and propose a *sequential simulation and estimation* account. We will use the interaction of motor, somatosensory, and auditory systems in speech production as an example to illustrate such internal cascaded processes, which can generalize to other sensory domains.

### Mental imagery of speech as sequential estimation

Perception and production systems are functionally connected: perceptual systems analyze the sensory input generated by self actions; the motor system is also regulated by perceptual feedback to perform updates on actions in the future. For example, when people talk, they move their articulators, feel the movement, and hear the self-produced speech that can be used to detect and correct any pronunciation errors. The temporal sequence of physical articulation, proprioception of the articulators, and auditory perception of one's own vocalization makes it possible—on the basis of co-occurrence and associative learning during development—to create internal connections among the neural processes that mediate motor action, somatosensory feedback, and auditory perception. After establishing the connections, motor commands can cycle internally through somatosensory regions and “reach” auditory regions. That is, the estimation in the somatosensory system can serve as a link between motor and auditory systems. Theoretically, such a cascaded estimation architecture has been hypothesized by Hesslow (2002). Anatomically and functionally, the connections between parietal regions and auditory temporal regions have also been demonstrated (Schroeder et al., 2001; Foxe et al., 2002; Fu et al., 2003).

**Figure 1:**
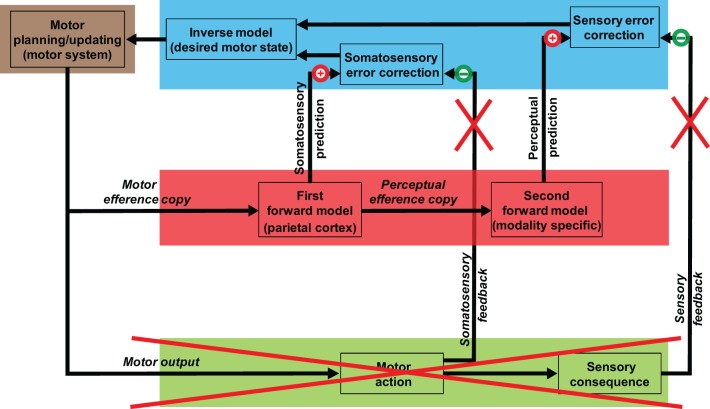
**Model of speech processing and its implication for mental imagery of speech.** The internal simulation and estimation model proposed as a second route to generate mental images. The motor systems that mediate action preparation carry out the same functions in mental imagery of speech, but only perform motor simulation, in the sense that the planned motor commands are truncated along the path to primary motor cortex and are not executed (the red cross over external outputs). A copy of such planned motor commands (motor efference copy) is processed internally and is used to estimate the associated somatosensory consequences. A copy of the somatosensory estimation is further sent to modality-specific areas, and the associated perceptual consequences that would be produced by the overt action are estimated. The quasi-perceptual experience during mental imagery (the feeling of movement of the articulators and the feeling of auditory perception in the case of articulation imagery) is the result of residual activity from these internal estimation processes, because of the absence of cancellation from the external feedback (the red crosses over external somatosensory and perceptual feedback).

On the basis of recent neurophysiological (MEG) studies, we proposed that a process of auditory inference after somatosensory estimation occurs during *overt speech processing* [Figure 1; adapted from Tian and Poeppel (2010)]. Specifically, the estimation of auditory consequences relies on the somatosensory estimation that derives from the simulation of planned action. That is, the internal *auditory* prediction is the result of a coordinate transformation from the somatosensory to the auditory domain. This sequential estimation mechanism (motor plan → somatosensory estimation → auditory prediction/estimation) can derive detailed auditory predictions that are then compared with auditory feedback for self-monitoring and online control.

In the case of the *mental imagery of speech*, we propose that the quasi-perceptual experience of articulator movement and the subsequent auditory percept are induced by the same sequential estimation mechanism. However, the “cancellation” deriving from somatosensory and auditory feedback, which is generated by the overt outputs during production, is absent in the imagery case (Figure 1). Therefore, similar to the case of motor imagery (Jeannerod, 1994, 1995), the feeling of articulator movement is the result of residual somatosensory representation resulting from motor simulation; the subsequent auditory perceptual experience, we suggest, is the residual auditory representation from the second estimation stage.

**Figure 2:**
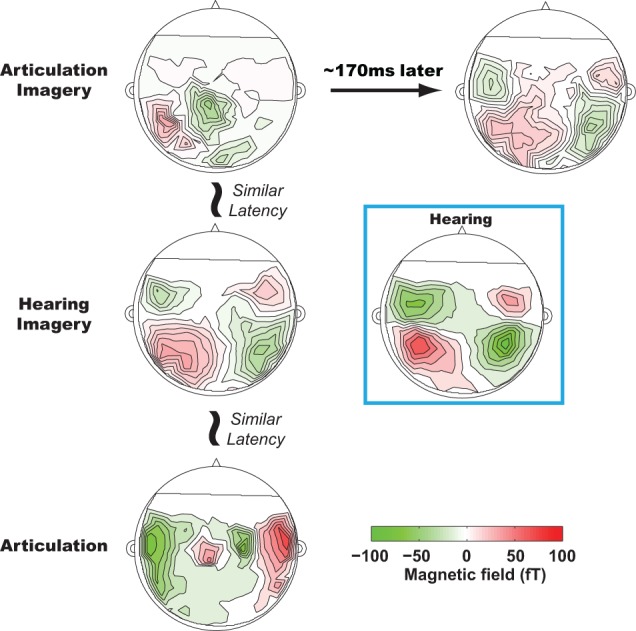
**Results from Tian and Poeppel (2010).** The sequential estimation during articulation imagery revealed by MEG recordings. All plots are MEG topographies (response patterns) when participants actually speak (lower row), imagine hearing (middle row), and imagine speaking (top row). The activity patterns in the first column are temporally aligned with the onset of articulation movement. At a similar latency, bilateral frontal, bilateral temporal, and left lateralized parietal activity patterns are observed in articulation, hearing imagery, and articulation imagery conditions. In articulation imagery, about 150–170 ms later after the parietal activity, bilateral temporal activity is also observed. All the bilateral temporal activity patterns in *hearing imagery* and *articulation imagery* resemble the topography of the auditory response during actual hearing (highlighted in a blue box, response pattern when participants listen to the same auditory stimuli as in other conditions).

On the basis of sequential estimation account, particular neural activity patterns for the two sequential estimates are predicted to occur in a temporal order. Specifically, an auditory pattern should follow a somatosensory one during mental imagery of speech. Applying a novel multivariate technique (Tian and Huber, 2008; Tian et al., 2011) to MEG data, we observed such a temporal order for somatosensory and auditory estimations during articulation imagery (Tian and Poeppel, 2010), manifested in the sequential activity patterns over modality-specific regions at different latencies (Figure 2). A left parietal response pattern was observed during *articulation imagery* at the same latency as when motor responses occurred in the articulation condition^1^. Following such a left parietal response pattern, a second pattern was identified at a latency of 150–170 ms after the parietal response. This second pattern was very similar to the response elicited by external auditory stimuli. Moreover, in a further experimental condition, *hearing imagery*, we also observed an auditory-like neural response pattern; however, its latency was faster than the same auditory pattern observed in *articulation imagery*. The existence of these two spatially highly similar auditory-like neural representations, with different latencies for *articulation* versus *hearing* imagery tasks, suggests that the same (or strikingly similar) neural representations can be generated either by internal estimation or by memory retrieval, based on contextual variation and task demands.

Note that the auditory estimation is presumably formed along the canonical auditory hierarchy, but the induction process will be in reversed order. That is to say, the abstract representation is (re-)constructed first in higher level associative areas and conveyed to a perceptual-sensory representation in lower areas. The observation of neural activity in the posterior superior temporal sulcus (pSTS) during silent speaking (Price et al., 2011) could be the result of an earlier reconstruction. Whereas the observations of similarity between responses to mental imagery and to external stimulation, such as in visual (e.g., Kosslyn et al., 1999) and auditory (e.g., Figure 2, Tian and Poeppel, 2010) domains, are the results of process continuation to lower perceptual-sensory regions. How much further back the reconstruction process might go seems to depend on the sensory modality and demands of the imagery tasks (Kosslyn and Thompson, 2003; Kraemer et al., 2005; Zatorre and Halpern, 2005).

## Internal simulation-estimation and relation to sensory-motor integration

**Figure 3:**
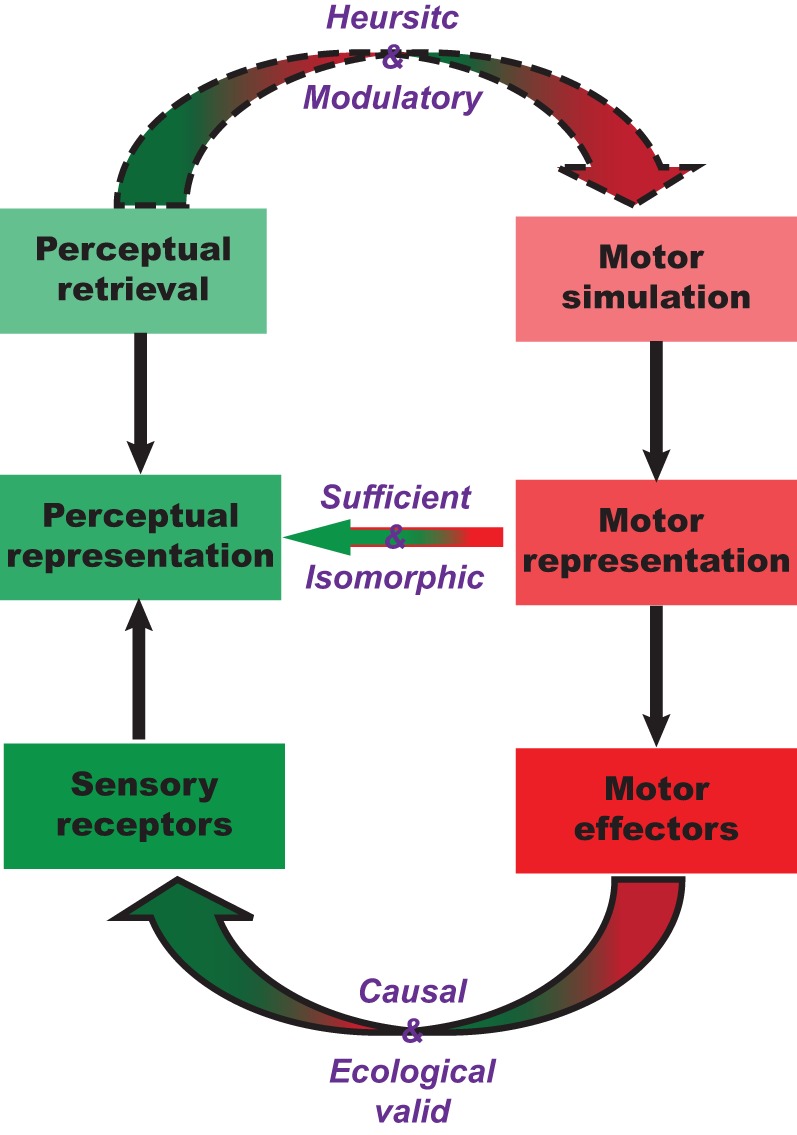
**Sufficiency and necessity between motor simulation and perceptual estimation.** The characteristics of the proposed motor simulation and perceptual estimation processes, and the nature of motor involvement during perceptual tasks. The internal motor simulation can take a similar path as motor preparation to derive a corresponding motor representation that in turn derives associated perceptual representations in a one-to-one fashion. Such one-to-one mapping is the same as the one in the external connections between the similar motor action and perceptual consequences. In the other direction, when the perceptual representation is needed, different paths can be taken. It can rely on memory retrieval to directly recreate the perceptual representation. It can also take another less demanding path that relies on the motor simulation to derive the associated perceptual representation.

Mental imagery of speech exemplifies a top-down mechanism for sensory-motor integration. The proposal here is motor simulation and sequential estimation. In the first part of this section, we describe the nature of this sequential transformation between motor, somatosensory, and other perceptual systems. We postulate that there is a one-to-one transformation between motor simulation and somatosensory estimation, as well as isomorphic mapping between somatosensory estimation and subsequent perceptual estimation (Figure 3). The entire transformation process is carried out in a continuous manner, beginning with motor simulation, then somatosensory estimation, and ending with modality-specific perceptual estimation. In the second part of this section, we argue that the implementation of motor simulation depends on context and task demands and may only exert modulatory effects on perception.

### Motor-to-sensory mapping: isomorphism via established connections

The central idea underpinning motor simulation and subsequent perceptual estimation is the conjectured *one-to-one mapping* or *isomorphism* between mental and physical processes. This isomorphism has been proposed for motor simulation (Jeannerod, 1994) and visual mental rotation (Shepard and Cooper, 1986): the intermediate stages of the internal process must have a one-to-one correspondence to intermediate stages of an actualized physical process. We extend this isomorphism to the associations between the motor simulation and perceptual estimation: the one-to-one mapping between the trajectory of motor simulation and perceptual estimation is a close analog to the causal relation between motor outputs and perceptual changes. That is, not only should the starting and ending points of an action simulation lead to the initiation and results of perceptual estimation, but intermediate points on this action simulation trajectory should result in a sequence of perceptual estimates, even though no external signals are physically presented. Notice that the analogy between internal simulation-estimation and external action-perception does not require the preservation of first-order isomorphism: only the one-to-one relation in the transformation of internal representation from motor to perceptual systems is required, as if the action was actually performed and the percept was actually induced.

The isomorphic transformation from motor to perceptual systems relies on the established internal associations between motor and perceptual representations, which are presumably formed following the causal and ecologically valid sequential occurrence of action-perception pairs, through the mechanisms of associative learning (Mahon and Caramazza, 2008). For example, the movement of articulators can induce somatosensory feedback and subsequent auditory perception of one's own speech. On the basis of the occurrence order (action first, then somatosensory activation, followed by auditory perception), an internal association can be established to link a particular movement trajectory of articulators with the specific somatosensory sensation, followed by a given auditory perception of speech. Note that we do *not* exclude the possible existence of a parallel estimation process that links motor simulation to somatosensory and auditory systems separately (Guenther et al., 2006; Price et al., 2011). Such an additional mechanisms which runs in parallel may mediate the early comparison between auditory estimation from an articulatory plan and intended auditory targets during speech production (Hickok, 2012). The redundancy of the compensation in somatosensory and auditory domains offers a hint for the co-existence of sequential and parallel estimation structures (Lametti et al., 2012). We suggest that the serial updating structure as one of the possible underlying estimation mechanisms naturally follows the biological sequences, providing advantages in learning and plasticity during development as well as online speech control.

Speech-induced suppression and enhancement caused by feedback perturbation provides strong evidence for the one-to-one mapping between motor simulation and estimation of perceptual consequences. When participants speak and listen to their own speech, the evoked auditory responses are smaller compared with the auditory responses to the same speech played back without spoken outputs (Numminen et al., 1999; Houde et al., 2002; Eliades and Wang, 2003, 2005; Ventura et al., 2009). However, when the auditory feedback is perturbed (manipulating, e.g., pitch or format frequencies), the auditory responses during speaking become larger compared with the ones during playback (Eliades and Wang, 2008; Tourville et al., 2008; Zheng et al., 2010; Behroozmand et al., 2011). The suppression caused by articulation demonstrates that an internal signal labels the onset of movement and down-regulates sensitivity to subsequent auditory perception (general suppression). However, the enhancement caused by feedback perturbation suggests that the internal signal during articulation is not a generic gain control mechanism for all auditory stimuli, but rather provides a precise perceptual prediction and only blocks the feedback that is identical to the prediction. In other words, there is a one-to-one mapping between motor simulation and auditory estimation, and the precise auditory consequence can be predicted based on particular motor trajectory.

The hypothesized intermediate neurocomputational step of somatosensory estimation that lies between motor simulation and auditory estimation has also been suggested by recent experiments. The sequential neural activity underlying somatosensory and auditory estimation has been observed during articulation imagery using MEG (Tian and Poeppel, 2010), as discussed above (Figure 2). Lesions over the left pars opercularis (pOp) in the inferior frontal gyrus (IFG) as well as adjacent to the left supramarginal gyrus (SMG) in parietal cortex correlate with the ability to imagine speech; this demonstrates the possible neural implementation underlying the proposed simulation and (somatosensory) estimation (Geva et al., 2011). Moreover, the causal role of somatosensory feedback in speech perception has also been demonstrated (Ito et al., 2009). There, participants were asked to listen to ambiguous stimuli (e.g., head-had vowel continuum) while their facial skin was manipulated with a robotic device. When the skin at the side of mouth was stretched upward (as in the case of pronouncing “head”), participants were biased toward hearing the ambiguous sound as “head.” That is, the somatosensory status affected the auditory perception in a systematic way: there was a one-to-one representational mapping between somatosensory and auditory systems.

### The simulation-estimation process in perception

The debates surrounding motor theories of perception and cognition [see the review by Scheerer (1984)] have heated up since the discovery of the putative “mirror neuron system” in monkeys (di Pellegrino et al., 1992; Gallese et al., 1996; see Rizzolatti and Craighero, 2004 for a review) and the observation of motor activity observed during numerous perceptual studies in humans (e.g., Rizzolatti et al., 1996; Iacoboni et al., 1999; Buccino et al., 2001; Wilson et al., 2004). Although these debates are beyond the scope of this review, the proposed mechanism of sequential estimation following motor simulation may provide insight to reconcile some of the observations, providing a top-down perspective.

We propose, building on arguments in the recent literature (Mahon and Caramazza, 2008; Hickok, 2009; Lotto et al., 2009; Rumiati et al., 2010), that the deployment of motor simulation in perceptual tasks is (1) strategy-dependent and (2) exerts modulatory effects on the formation of perceptual representations. That is, the selection of motor involvement in perceptual tasks depends on context and task demands. It is a top-down strategic step to provide modality-specific representations in advance (cf. Moulton and Kosslyn, 2009) and reduce perceptual variance by generating more precise estimation (Mahon and Caramazza, 2008; but also see Pulvermüller and Fadiga, 2010 for an opposite view from a embodied perspective).

#### The implementation of motor-to-sensory transformations is strategy-dependent

We describe two types of evidence. First, the recruitment/involvement of motor simulation is influenced by task demands. For example, motor imagery can be performed from a “first person” perspective that relies on kinesthetic feeling, in contrast with when a task is executed from a “third person” perspective in which the action-related visual changes are recreated (Jeannerod, 1994, 1995). Reaction times of hand rotation imagery showed an interaction between imagery perspectives and limb posture: when asked to imagine rotating their hands from first person perspective, participants responded faster when their hands were on the lap but slower when their hands are in the back; the reverse pattern was observed when imagining from third person perspective (Sirigu and Duhamel, 2001). Activation in the motor system was observed when participants were explicitly told to imagine rotating an object with their own hands, but was absent when they were told to imagine rotating the same object with a robotic motor (Kosslyn et al., 2001). Both behavioral and neuroimaging results highlight that the task demands influence the implementation of neural pathways that mediate either direct simulation (memory retrieval) or motor simulation-estimation (transformation between motor and perceptual systems).

Second, motor-to-sensory transformations are influenced by context and the properties of stimuli. For example, neural responses in frontal motor regions have been observed during observation of meaningful actions, contrasted with occipital activity for meaningless actions (Decety et al., 1997). Relatedly, when participants mentally rotated their hands, premotor, primary motor, and posterior parietal cortices were activated. However, frontal motor areas were silent when they mentally rotated objects (Kosslyn et al., 1998). These results suggest that contextual influence and task demands can determine the implementation of motor simulation in a top-down, voluntary, strategic way.

In the context of action observation, understanding/comprehension and imitation could be the result of heuristic engagement of motor simulation. That is, humans can deploy a top-down mechanism that transfers perceptual goals into the motor domain and initiates motor simulation to derive perceptual consequences (Figure 3). The strategic and heuristic initiation of motor involvement can be considered as a top-down mental imagery process (possibly exclusive to humans) (cf. Iacoboni et al., 1999; Papeo et al., 2009), wherein the motor action is internally simulated and perceptual consequences estimated thereafter (cf. Tkach et al., 2007).

#### Modulatory function of motor simulation on perception

The major evidence supporting a modulatory role of motor simulation in perception (rather than a primary causal role) comes from lesion studies. For example, lesions in the frontal lobe only caused deficits in action production, whereas lesions in the parietal lobe caused deficits both during production and perception of movement (Heilman et al., 1982). A deficit in gesture recognition has also been linked to inferior parietal cortex lesions but not lesions in the frontal lobe (Buxbaum et al., 2005). Action comprehension also relies on a network that includes inferior parietal cortex but not IFG (Saygin et al., 2004). Although patients with IFG lesions demonstrated deficits in action comprehension in the same study, the static stimuli (pictures of pantomimed actions or objects) could require participants to implement the strategy of motor simulation to form the dynamic display of action and to derive the perceptual consequences so that they can fulfill the action-object association task. Such lesion results indicate that a damaged motor system (and the deficits in motor simulation) dissociates from action-perception and comprehension. The abstract meaning of motor action is probably “stored” in parietal regions, and the motor simulation mediated by frontal regions is one of many paths to access the stored representation (in line with our proposed *simulation* over frontal cortex and *estimation* over parietal cortex). Therefore, motor simulation to estimate perceptual consequences is only modulatory and not necessary for perceptual tasks.

Analogous to the advantage of multisensory integration in minimizing perceptual variance (Ernst and Banks, 2002; Alais and Burr, 2004; van Wassenhove et al., 2005; von Kriegstein and Giraud, 2006; Morgan et al., 2008; Poeppel et al., 2008; Fetsch et al., 2009), the modulatory effects of motor simulation convey benefits by providing additional, more detailed information to enrich the perceptual representation using internal sequential estimation mechanism (cf. Mahon and Caramazza, 2008). Human observers can adopt motor strategies to provide more precise perceptual representations and deal with perceptual ambiguity, for example in the case of speech perception. That is, the motor simulation and estimation can provide improved priors to reduce perceptual variance.

In summary, various perceptual tasks can use the motor system to derive perceptual consequences, by implementing the same top-down motor simulation and perceptual estimation mechanism, as in mental imagery of speech. We hypothesize that this motor simulation is modulatory and only serves as one of many possible corridors to induce perceptual representations. Such strategies of sensory-to-motor and motor-to-sensory transformation would be implemented depending on task demands and contextual influence.

## Implications for the neural correlates of some disorders

In this section we argue that the internal processes of motor simulation and estimation, revealed originally for the mental imagery of speech, can shed light on possible neural correlates of certain disorders, including auditory hallucinations, stuttering, and phantom limb syndrome. We outline some working hypotheses regarding these disorders, complementing other existing hypotheses. It is suggested that the proposed idea for mental imagery generation, motor simulation, and sequential perceptual estimation, points to the practical value of mental imagery research for understanding the internal mechanisms of such neural disorders.

### Auditory hallucinations: intact estimation versus broken monitoring

Internal simulation and sequential estimation has been proposed to be a way to distinguish between the perceptual changes caused by self-generated actions and exogenous external events (Blakemore and Frith, 2003; Jeannerod and Pacherie, 2004; Tsakiris and Haggard, 2005). The perceptual consequences of intended movement can be predicted, and the processing of external sensory feedback can be dampened by the internal prediction, such as in the case of speech production (e.g., Houde et al., 2002; Eliades and Wang, 2003, 2005) and somatosensory perception in tickling (e.g., Blakemore et al., 1998). This suggests that the action-induced perceptual signals are identified as self-generated and cancelled by the virtually identical representation generated by internal perceptual prediction. However for patients suffering from auditory hallucinations, deficits of these hypothesized dampening mechanisms for self-induced perceptual changes have been observed in both somatosensory (e.g., Blakemore et al., 2000) and auditory (e.g., Ford et al., 2007; Heinks-Maldonado et al., 2007) domains. These results suggest that patients with auditory hallucinations cannot separate self-induced from external-induced perceptual signals.

Critically, deficits of distinguishing self-induced from externally induced perceptual changes are not enough to account for auditory hallucinations, because the positive symptoms typically occur in the absence of any external stimuli. There must exist an internal mechanism to induce the auditory representations that are then misattributed to an external source/voice. In fact, we face a similar situation during mental imagery: the neural representations mediating perception and mental imagery are very similar, but there is no mechanism in the perceptual system to distinguish them. A *source monitoring function* is required to keep track of the origins of the perceptual neural representation. Therefore, we hypothesize that a higher order function monitors and distinguishes internally versus externally induced neural representations. Such a monitoring operation is functionally independent from the perceptual estimation process that internally reconstructs the perceptual representation. Under this hypothesis, auditory hallucinations are caused by incorrect operation of the monitoring function, resulting in incorrectly labeling the self-induced auditory representation during the intact internal perceptual estimation processes.

Computationally, the independence of the monitoring function versus internal simulation and estimation is demonstrated by the nuanced differences between *corollary discharge* and the *efference copy* [see the review by Crapse and Sommer (2008)]. The efference copy is a duplicate of the planned motor command and provides the dynamics of an action trajectory that can be used to estimate the perceptual consequences (von Holst and Mittelstaedt, 1950, 1973). Corollary discharge is a more general motor related mechanism that can be available at all levels of a motor process. The corollary discharge does not necessarily contain the same representational information as an efference copy; rather, it serves as a generic signal to inform sensory-perceptual systems of the potential occurrence of perceptual changes caused by one's own actions (Sperry, 1950). In the case of speech articulation, these two functions originate at the same stage of motor simulation, but their functional roles are still separate. The efference copy is used to estimate the detailed perceptual consequences, whereas the corollary discharge labels the internally and externally induced perceptual consequences.

Empirically, the finding that auditory hallucination patients can generate inner speech (e.g., Shergill et al., 2003) demonstrates the relatively intact motor-to-sensory transformation function. The neural responses in IFG and superior temporal gyrus/sulcus (STG/STS) were observed during auditory hallucinations, hinting at the derivation of auditory perceptual consequences from motor simulation during the positive symptom (e.g., McGuire et al., 1993; Shergill et al., 2003). Moreover, the left lateralization during covert speech versus right lateralization during auditory hallucinations offers tantalizing hints about the independence between self-monitoring and the sequential simulation-estimation (Sommer et al., 2008).

We summarize the hypothetical mechanistic account for auditory hallucinations (of this type) as follows: when patients prepare to articulate speech covertly or subvocally (either consciously or unconsciously), the internal motor simulation leads to perceptual estimation (intact efference copy). But the source monitoring process malfunctions (broken corollary discharge). Therefore, the internal prediction of a perceptual consequence, which has the same neural representation as an external perception, is erroneously interpreted as the result of external sources, resulting in an auditory hallucination.

### Stuttering: noisy estimation and correction processes

The comparison between internal estimation and external feedback provides information to fine-tune motor control. However, if the internal estimate from motor simulation malfunctions and generates imprecise perceptual predictions, an inaccurate or incorrect feedback control signal would be conveyed. Stuttering could be an example of such erroneous correction. We suggest, along the lines of similar theories (Max et al., 2004; Hickok et al., 2011), that one of the neural mechanisms causing stuttering is a deficit in the motor-to-sensory transformation. That is, the noisy perceptual estimation is mismatched to the external feedback. Such a discrepancy would signal an incorrect error message, and the feedback control system would interpret such an apparent error as the requirement to correct motor action. Hence, unnecessary attempts would be performed to modify the correct articulation, resulting in repetitive/prolonged sound or silent pauses/blocks.

The noise in the estimation process can come both from the somatosensory and auditory domains (since there is sequential estimation). Stutterers showed speed and latency deficits when required to sequentially update articulator movement (Caruso et al., 1988). Smaller magnitude compensation with longer latency adjustment to the perturbation on the jaws was also observed in stutterers (Caruso et al., 1987). In the auditory domain, smaller magnitude compensation to the perturbation of F1 formant in auditory feedback is observed (Cai et al., 2012). The inaccurate compensation to external perturbation in both somatosensory and auditory domains (with intact somatosensory and auditory processes) demonstrates that inaccurate prediction in both domains could be causal for stuttering.

Interestingly, dramatically altering auditory feedback (e.g., by delaying feedback onset or shifting frequency) can enhance speech fluency in people who stutter (Martin and Haroldson, 1979; Stuart et al., 1997, 2008). The improvement could be because the magnitude of error signals is scaled down when the distance between feedback and prediction is beyond some threshold, so that fewer correction attempts are made.

### Phantom limbs: mismatch between internal estimation and external feedback

The mismatch between internal prediction and external feedback could also be caused by an acute change of conditions leading to the absence of feedback. One such example is the phantom limb phenomenon, where amputees feel control over a lost limb (phantom limb) accompanied with chronic and sometimes acute pain. We hypothesize that the apparent awareness and control of a lost limb occurs as follows: the missing somatosensory feedback is “replaced” by the results of internal estimation (cf. Frith et al., 2000; Fotopoulou et al., 2008). Such a hypothesis is similar to the mislabeling of the internal estimation as an external perception (due to the malfunction of source monitoring) in auditory hallucinations.

The causes of pain in phantom limbs are more intriguing. The most significant physical changes are loss of proprioception, or somatosensory afference, after lost limbs. Because motor control as well as motor simulation of the lost limb are still in some sense valid (e.g., Raffin et al., 2012), we hypothesize that a mismatch between the intact internal estimation and absent external somatosensory feedback can cause the pain associated with phantom limbs. In fact, consistent with our hypothesis, limb pain can be induced in normal participants by mismatching visual and proprioceptive feedback (McCabe et al., 2005) and spinal cord injured patients report that neuropathic pain increases while they imagine moving their ankles (Gustin et al., 2008).

This mismatch hypothesis may represent an intermediate step between cortical reorganization and pain induction. Lost limbs cause reorganization in both motor (Maihöfner et al., 2007) and somatosensory (Maihöfner et al., 2003) cortices, and pain reduction has been demonstrated to correlate with more granular organization in the same areas (MacIver et al., 2008). Motor imagery can lead to cortical reorganization that correlates with pain reduction in phantom limbs (Moseley, 2006). Seeing the movement of the opposite functioning arm in a mirror can reduce the pain associated with the phantom limb (Ramachandran et al., 1995). Such behavioral and psychological training can provide more precise topographic maps in both motor and somatosensory cortices and hence reduce the inaccurate motor firing caused by the “take over” effect (e.g., cortex of lip movement expand to the cortex mediated a lost hand), as well as erroneous somatosensory estimation. The internal estimation hypothesis offers a new perspective on pain induction. However, there is neither a clear pain center (Mazzola et al., 2012) nor a mechanistic pain induction account (Flor, 2002). Further research is needed to understand how the proposed mismatch hypothesis could underpin pain induction.

## Conclusion

In this perspective, we argued that mental imagery is an internal predictive process. Using mental imagery of speech as an example, we demonstrated a variety of principles underlying how the mechanism of motor simulation and sequential perceptual estimation in mental imagery works. We conclude that the simulation-estimation mechanism provides a novel conceptual and practical perspective that allows for new types of research on predictive functions and sensory-motor integration, as well as stimulating some new insights into several neural disorders. Typically, mental imagery has been studied in cognitive psychology and cognitive neuroscience, while the concepts of internal forward models (and sensory-motor integration) are the focus of motor control research from an engineering perspective. Our atypical pairing of internal models as an additional source for mental imagery yields, in our view, some provocative new angles on mental imagery in both basic research and applied contexts.

### Conflict of interest statement

The authors declare that the research was conducted in the absence of any commercial or financial relationships that could be construed as a potential conflict of interest.

## Footnotes

^1^Because previous findings suggest that the time courses for completing execution and imagery are comparable (Decety and Michel, 1989; Decety et al., 1989; Sirigu et al., 1995, 1996), the observed neural responses over parietal regions presumably mediate somatosensory estimation.
